# The Neural Correlates of Spontaneous Beat Processing and Its Relationship with Music-Related Characteristics of the Individual

**DOI:** 10.1523/ENEURO.0214-24.2024

**Published:** 2024-10-17

**Authors:** Alyssa C. Scartozzi, Youjia Wang, Catherine T. Bush, Anna V. Kasdan, Noah R. Fram, Tiffany Woynaroski, Miriam D. Lense, Reyna L. Gordon, Enikő Ladányi

**Affiliations:** ^1^Department of Otolaryngology - Head & Neck Surgery, Vanderbilt University Medical Center, Nashville, Tennessee 37203; ^2^Vanderbilt Genetics Institute, Vanderbilt University Medical Center, Nashville, Tennessee 37203; ^3^College of Medicine, University of Illinois Chicago, Chicago, Illinois 60612; ^4^Department of Hearing and Speech Sciences, Vanderbilt University Medical Center, Nashville, Tennessee 37203; ^5^Department of Linguistics, University of Potsdam, Potsdam 14476, Germany

**Keywords:** beat processing, brain–behavior correlations, musicality, neural oscillations

## Abstract

In the presence of temporally organized stimuli, there is a tendency to entrain to the beat, even at the neurological level. Previous research has shown that when adults listen to rhythmic stimuli and are asked to imagine the beat, their neural responses are the same as when the beat is physically accented. The current study explores the neural processing of simple beat structures where the beat is physically accented or inferred from a previously presented physically accented beat structure in a passive listening context. We further explore the associations of these neural correlates with behavioral and self-reported measures of musicality. Fifty-seven participants completed a passive listening EEG paradigm, a behavioral rhythm discrimination task, and a self-reported musicality questionnaire. Our findings suggest that when the beat is physically accented, individuals demonstrate distinct neural responses to the beat in the beta (13–23 Hz) and gamma (24–50 Hz) frequency bands. We further find that the neural marker in the beta band is associated with individuals’ self-reported musical perceptual abilities. Overall, this study provides insights into the neural correlates of spontaneous beat processing and its connections with musicality.

## Significance Statement

Humans possess the remarkable ability to perceive the beat in musical pieces, with neural oscillations suggested to play a key role. While most previous research has focused on beat processing during active attention to stimuli, the mechanisms underlying spontaneous beat perception remain less understood. Furthermore, the link between neural markers of beat processing and both self-reported musicality and behavioral measures of beat perception has been scarcely investigated. Our study demonstrates that spontaneous beat processing shares similar neural correlates with active listening when the beat is acoustically marked. Moreover, our results indicate a connection between these neural markers and self-reported musicality, expanding our understanding of the neural bases of musical rhythm perception.

## Introduction

Humans can extract the beat of musical pieces, even spontaneously. The beat is the perceived pulse or tempo of a musical piece, and a metrical structure is created based on the organization of stronger (aka downbeat) and weaker beats by the listeners. The position of the beat and the metrical structure depends on both the physical characteristics of the musical stimulus and the individual's interpretation. If there is no strong beat determined by physical cues (i.e., accent, grouping, melody), individuals can even consciously change the position of the beat (Iversen et al., 2009; Nozaradan et al., 2011, 2012).

The role of neural oscillations in metrical processing has been emphasized by several previous studies (Stefanics et al., 2010; Bouwer and Honing, 2015; Doelling et al., 2019; Bouwer et al., 2023). Entrainment theories propose that the phase and period of neural oscillations synchronize with rhythmic stimuli of the environment at various frequencies, so that neural excitability will be the highest at the expected time points in the stimuli, leading to optimal processing of auditory stimuli (see Large et al., 2015; Haegens and Zion Golumbic, 2018 for reviews).

Oscillations in the beta (13–23 Hz) and gamma (24–50 Hz) frequency bands have been found to be associated with metrical processing (Zanto et al., 2005, 2006; Fujioka et al., 2009, 2012, 2015; Iversen et al., 2009; Kasdan et al., 2022; Persici et al., 2023). Iversen et al. (2009) presented participants with a repeating tone-tone-rest pattern in blocks and participants were either asked to imagine the beat on the first or the second tone, or the beat was physically accented with an increased intensity. Participants showed an increased beta response to the beat both when the beat was imagined and physically accented, but the gamma response was increased at the beat only when it was physically accented. The authors propose that the results can be accounted for by the involvement of the motor system in metrical interpretation. Increased power in the beta and gamma frequency bands to physically accented beats have been found both when participants pay attention to the stimuli (Iversen et al., 2009) and when they do not (Kasdan et al., 2022; Persici et al., 2023). It is, however, unknown if the increased evoked beta activity in the case of physically not accented beats is associated with attention to the signal or if it also appears during passive listening.

Individual differences in the characteristics of neural oscillations measured during beat processing may be linked to behavioral correlates of beat processing. Previous research has found that individual differences in oscillatory measures of beat processing are correlated with tapping performance (Nozaradan et al., 2016). Similarly, we expect higher musicality or musical sophistication, which accounts for musical aptitude, interest, and training, to be related to the neural correlates of beat processing. There is previous evidence of the relationship of musical sophistication with oscillatory measures of beat processing, namely, the auditory steady-state response (Manting et al., 2021).

The present study investigates the processing of simple beat structures when the beat is physically accented and when it is inferred from a previously presented accented beat structure. While previous work using a similar paradigm (Iversen et al., 2009) relied on a limited sample size, our study involves a considerably larger sample. Moreover, while previous studies have predominantly focused on the neural correlates of beat processing when participants are instructed to actively pay attention to the stimuli or to imagine beat patterns, we aim to examine beat processing in a more naturalistic context. Specifically, we assess the neural mechanisms underlying spontaneous beat perception by exposing participants to auditory stimuli without explicit instructions, thereby mirroring real-world listening experiences more closely. Additionally, we aim to examine the relationship between neural correlates of accented and inferred beat processing with behavioral measures of rhythm discrimination and self-reported musicality. While past studies have often categorized musicality as a binary variable, focused on comparing musicians and nonmusicians, in the current study, we follow a more contemporary view that recognizes musicality as a multifaceted and nuanced trait that exists along a spectrum and can be quantified continuously (Müllensiefen et al., 2014).

Following previous work on the role of neural oscillations in beat processing, we hypothesized that physically accented beats would be associated with increased power in the beta and gamma frequency bands, and that inferred beats would be associated with an increased power only in the beta band. Furthermore, we hypothesized that there would be a positive relationship between neural responses to beat patterns and self-reported musicality as well as behavioral rhythm discrimination.

## Materials and Methods

### Participants

We analyzed data from 57 participants (54 female; mean age = 35.51 years; SD = 4.34 years: range, 27–47 years; handedness: right, 47). We tested an additional 13 participants but excluded them from analyses based on self-reported hearing impairment or self-reported neurological or genetic disorder (*n* = 7) or insufficient data quality (*n* = 6). Participants were drawn from a larger study on several aspects of parent and child language and music abilities. The participants in the present study are predominantly female because most participating parents were women, although we did not impose any constraints on the biological sex of the participating parents. We report data only from the parents in the current paper; we will report further data in future publications.

Participants completed (1) a behavioral rhythmic discrimination task, (2) an electroencephalography (EEG) beat perception task, (3) a musicality questionnaire, and (4) a neurological screening form and a demographic survey, in that order. The study was approved by the Vanderbilt University Medical Center Institutional Review Board, and participants provided informed consent in accordance with the Institutional Review Board policies. Upon completion of the study, participants were compensated for their time. Data were managed in a secure web platform for building and managing databases and surveys (Harris et al., 2009).

### Beat-based advantage task

The beat-based advantage (BBA) paradigm is a rhythmic discrimination task that assesses beat processing skills (Grahn and Brett, 2007, 2009). In each trial, participants were presented with a series of three rhythms, each ranging between 2.9 and 3.5 s, where the first two rhythms were identical. In half of the trials, the third rhythm was different from the first two, and, in the other half, the third rhythm was identical. Participants were asked to decide if the third rhythm was the same or different from the first two. In each trial, the rhythms were separated by 1,500 ms of silence. We used a version of the task with 16 trials (stimulus details are reported in Fiveash et al., 2022). Eight trials were simple rhythms, which had a clear strong beat, and 8 trials were complex rhythms, which did not have a clear beat. Stimuli were presented in a fixed order for all participants in order to reduce potential variability resulting from different item orders and to obtain a more accurate measurement of individual differences in rhythm discrimination skills.

Participants completed two practice items, in which they were allowed to listen to the stimuli any number of times; test items were presented only once. We instructed participants not to move or tap along with the rhythms. The volume level in the task was preset to a predetermined comfortable level; however, participants were allowed to adjust the volume level during the practice trials if needed. The task took 5–10 min to complete. The task instructions, stimuli, and responses were presented and recorded in REDCap, the web platform we also used for data management (Harris et al., 2009). Fifty-two participants provided valid data for the task. Forty participants completed the task in the lab, while 12 participants completed the task remotely (Total scores on simple, complex and overall d’ did not significantly differ between participants who completed the task remotely and those who completed the task on site (simple: *t*_(50)_ = 1.477, *p* = 0.146; complex *d*’: *t*_(50)_ = −1,654, *p* = 0.104, overall *d*’: *t*_(50)_ = 0.224, *p* = 0.826). Due to these differences, all analyses on associations between the BBA and EEG measures were conducted both with including and excluding participants who performed the online version of the task. As excluding participants who performed the task online did not change the results, we decided to report results of the analysis that included these participants). BBA data of one participant were excluded from analysis because they reported an intimate familiarity with Morse code, which caused them to perceive the rhythmic stimuli as letters and symbols instead of pure rhythms without semantic information.

Task performance was calculated for all trials, simple trials alone, and complex trials alone. We used signal detection analysis to calculate *d*’ as an index of rhythm discrimination performance (Macmillan and Creelman, 2005). We counted correct “different” responses on different trials as hits and incorrect “different” responses on the same trials as false alarms and used the difference between their *z*-scored value [*d*’ = *z*(hit rate) − *z*(false alarm)].

### Goldsmiths Musical Sophistication Index (questionnaire)

All participants completed the Goldsmiths Musical Sophistication Index (Gold-MSI; Müllensiefen et al., 2014), a self-report measure of musical sophistication. The Gold-MSI consists of 38 items, scored on a seven-point Likert scale, and measures six dimensions of musical sophistication. For the present study, we focused on three of these dimensions for subsequent analyses on associations with neural data: general sophistication, perceptual ability, and musical training. The Gold-MSI was implemented electronically in REDCap, and all participants completed the survey in the lab.

### Beat perception EEG task

**Figure 1.**
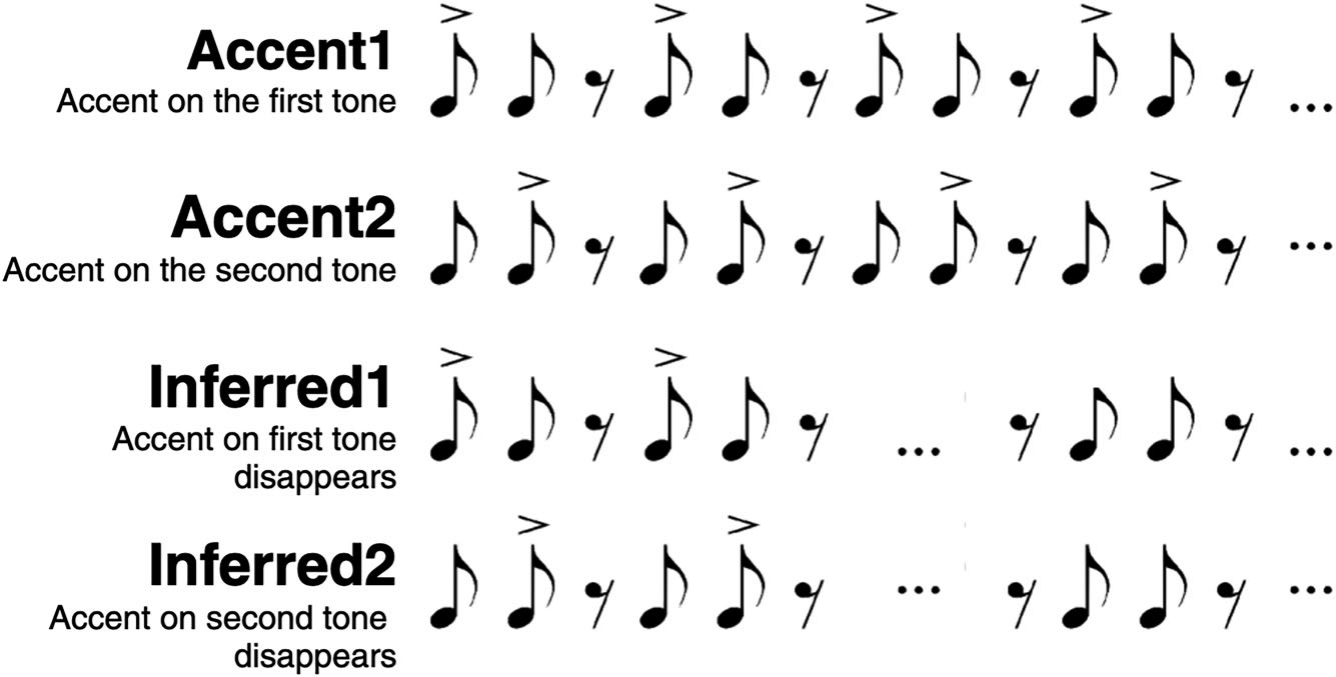
Stimuli used in the beat perception task. Figure adapted from Kasdan et al. (2022).

We adapted the beat perception task from Iversen et al. (2009). Participants were presented with a continuous series of sequences featuring two tones (woodblock sounds) followed by a rest. Tones had an approximate duration of 60 ms and a frequency of 656 Hz. Tones and rest were presented with an interonset interval of 200 ms. Each sequence lasted for 600 ms and was grouped into blocks of 50 sequences lasting 30 s total. Blocks were differentiated into four conditions: two physical accent conditions (Accent1, Accent2) and two inferred beat conditions (Inferred1, Inferred2). In the Accent1 and Accent2 conditions, physical accents were marked with an increase of approximately 9 dB in amplitude, creating an emphasis on either the first or the second tone. In the Accent1 condition, the accent was placed on the first tone, and in the Accent2 condition, the accent was placed on the second tone. The Inferred1 and Inferred2 conditions began with a physical accent on either the first or second tone. After the 10th sequence, the physical accent disappeared, and the two tones were presented at equal amplitude for the remaining sequences. We analyzed only sequences past the 10th in each condition. This design mirrors the design used by Iversen et al. (2009) except that in our study participants were not explicitly instructed to imagine a beat or pay attention to the auditory stimuli. Participants were presented with five blocks of each of the four conditions in a random order. During the task, participants watched a silent cartoon on a tablet screen (*The Snowman*, 1982, directed by Jackson and Murakami). Stimuli were played at 70 dB and presented over headphones (Sennheiser HD 280 PRO). The full beat perception task lasted approximately 10 min (Fig. 1).

### EEG acquisition

We completed the EEG recording sessions onsite at the Vanderbilt Music Cognition Lab. We recorded EEG data with Net Station Acquisition (v 5.4.2) using a high-density, geodesic array of 128 Ag/AgCl electrodes embedded in soft sponges (HydroCel GSN 130, EGI, Eugene) connected to a Net Amps 400 amplifier. Data were collected at 500 Hz with a high-pass 0.1 Hz online filter and referenced to the vertex (electrode Cz) during recording. The local impedance at each electrode was reduced by applying a KCl solution and was measured to be below 50 kΩ before the start of EEG recording.

### EEG preprocessing

We preprocessed EEG data in MATLAB (2020b) with the EEGLab toolbox (v2019.1, Delorme and Makeig, 2004). Channels 125–128, located around the facial muscles, were first removed from the data and excluded from all analyses due to their general noisiness. Data were then bandpass filtered from 0.5 to 100 Hz (zero-phase, noncausal finite impulse response filter), and a 60 Hz line noise was removed using the cleanLineNoise function from the PREP pipeline (Bigdely-Shamlo et al., 2015). Noisy channels were then identified with the Artifact Subspace Reconstruction (ASR) function (Chang et al., 2018) and interpolated. Continuous data were then cleaned using the ASR function with a burst criterion threshold of 20, and channels were re-referenced to the average of all electrodes. Next, independent component analysis was conducted to identify and remove EEG components associated with eye and cardiac activity, using the ICLabel tool in EEGLab (Pion-Tonachini et al., 2019). Components above the 70% threshold were identified and rejected after manual inspection. For each task condition, epochs were created from −400 to 1,200 ms from the onset of the first beat. Epochs beyond the ±100 µV threshold were rejected, and data were bandpass filtered from 0.5 to 55 Hz. An average of 2.92% of epochs were rejected from the whole sample, with an average of 194.16 (SD = 8.51) out of the 200 remaining across all four conditions. We had 194.57 (SD = 8.28), 194.12 (SD = 9.47), 193.84 (SD = 7.36), and 194.09 (SD = 9.00) epochs remaining for the Accent1, Accent2, Inferred1, and Inferred2 conditions, respectively.

### EEG data analysis

#### Time–frequency analysis

We conducted a wavelet-based time–frequency decomposition with the FieldTrip toolbox (Oostenveld et al., 2011) in MATLAB (2020b). Event-related potentials were generated from the average across sequences for each condition and used for evoked time–frequency analysis. Evoked (phase-locked) time–frequency representation was obtained for each condition by convolving the average ERP with a family of Morlet wavelets from 12 to 50 Hz, with a frequency step of 1 Hz and a time step of 2 ms. The width of the wavelets was five cycles, and we applied them in the time window (−200, +800), with a 0 being the onset of each tone-tone-rest pattern and a 200 representing the second tone in the sequence. Time–frequency representations (TFRs) were normalized at each time point, frequency, and channel to control for differences in absolute power between participants. We computed participant-specific average power matrices as baseline power per frequency and channel across all four conditions and across all time points. Using these participant-specific power matrices, we normalized our time–frequency data by computing the relative power change [(power − baseline power)  / baseline power) for each condition for each participant.

#### Cluster-based permutation statistical analyses

To test for differences in power between Accent1 versus Accent2 and Inferred1 versus Inferred2 conditions, we applied nonparametric cluster-based random permutation tests coded for within-subjects design, using the FieldTrip Toolbox (Oostenveld et al., 2011) in MATLAB (2020b). All statistics were performed on stimuli with nonoverlapping time windows and divided into the following frequency bins: beta (13–23 Hz) and gamma (24–50 Hz) in line with a study by Persici et al. (2023). The Accent1 versus Accent2 conditions were compared in the time window (−100, +500) at each time point and channel, generating clusters from adjacent channels and time points where the *t*-values were significant at *p *< 0.025 (two-tailed). The distance between neighboring channels was determined using the FieldTrip triangulation parameter, and one channel was required to have a minimum of two neighboring channels to be clustered together. The significance of each cluster was assessed using the Monte Carlo method by comparing the data from the actual cluster to 5,000 simulated “dummy” clusters of randomly permuted values from both conditions (Oostenveld et al., 2011); the resulting cluster *p*-values are the proportions of permutations in which the true cluster statistically exceeds the simulated “dummy” cluster statistic; *p *< 0.05 was considered significant. The same procedure was used to compare the inferred beat conditions (i.e., Inferred1 vs Inferred2) in the beta and gamma frequency bins.

#### Correlation between clusters and behavioral measures

We computed Pearson’s correlations examining the relationship between our behavioral measures and the mean power difference of the two conditions (i.e., Accent1 vs Accent2 or Inferred1 vs Inferred 2) from any significant clusters. Significant correlations were examined further with multiple linear regression models by adding age, socioeconomic status (as determined by self-reported education), and biological sex as covariates. Analyses were conducted using the cor.test() and lm() functions in R version 3.6.2.

## Results

**Figure 2.**
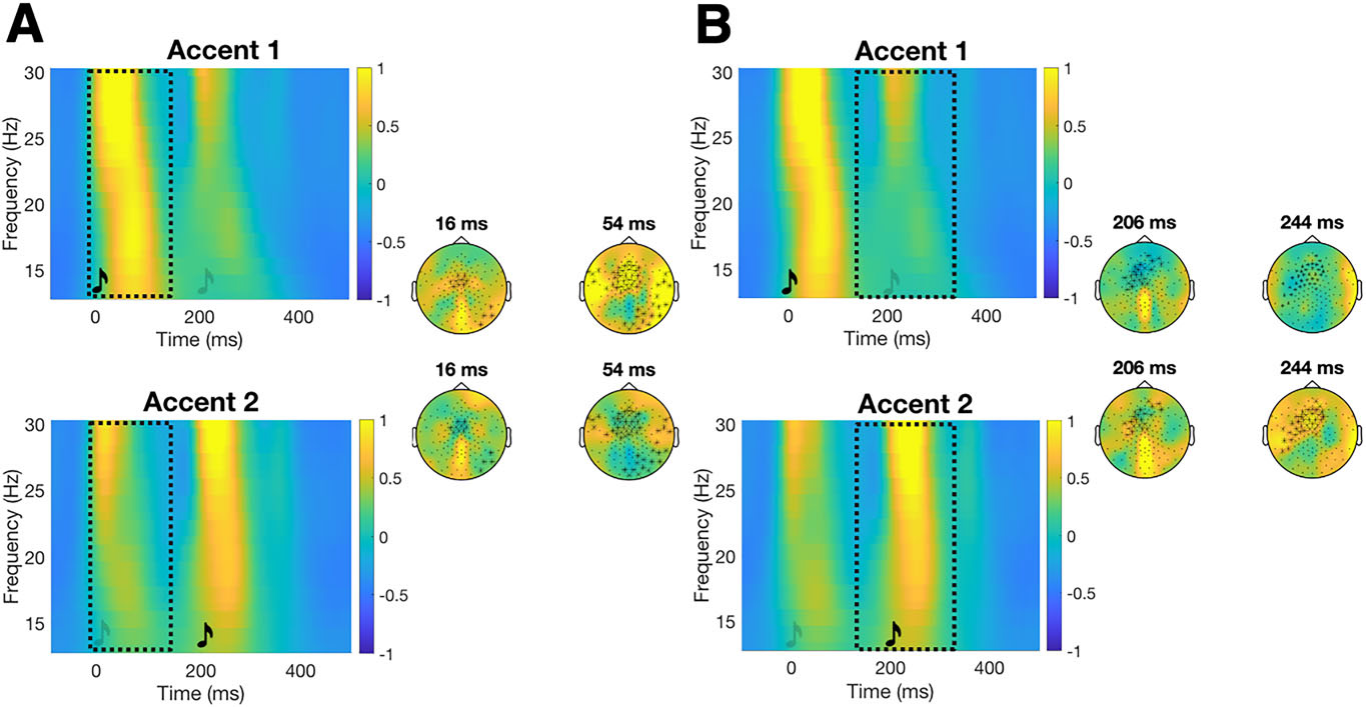
Accented-Beat1 (***A***) and Accented-Beat2 (***B***) beta effects. In the Accent 1 condition, the first tone was accented, and in the Accent 2 condition, the second tone was accented (see Fig. 1 for visual representation). An Accented-Beat1 effect shows a stronger neural response to the beat in the first tone, and an Accented-Beat2 effect shows a stronger neural response to the beat in the second tone (see Materials and Methods). Time–frequency representations (TFRs) and topographies for *N *= 57, showing baseline corrected power changes in EEG beta neural activity at the first tone (***A***, Accented-Beat1 effect) and at the second tone (***B***, Accented-Beat2 effect). TFRs are shown on the left side of each panel displaying the average of all the channels belonging to the cluster; the black dashed box indicates the time and frequency boundaries of the significant clusters. Representative topographies (on the right side of each panel) are shown at the indicated latencies within the clusters. The color scale represents the percent change from the baseline. The same color bar applies to both the TFR and the corresponding topo plots. Significant channels are marked with black asterisks. Increased beta neural activity (i.e., bright yellow on the color scale) is found at the first tone when this was accented [in the Accent1 condition (top row) as compared with the Accent2 condition (bottom row)] in ***A***, and increased beta neural activity is found at the second tone when this was accented [in the Accent2 condition (bottom row) as compared with the Accent1 condition (top row)] in ***B***.

**Figure 3.**
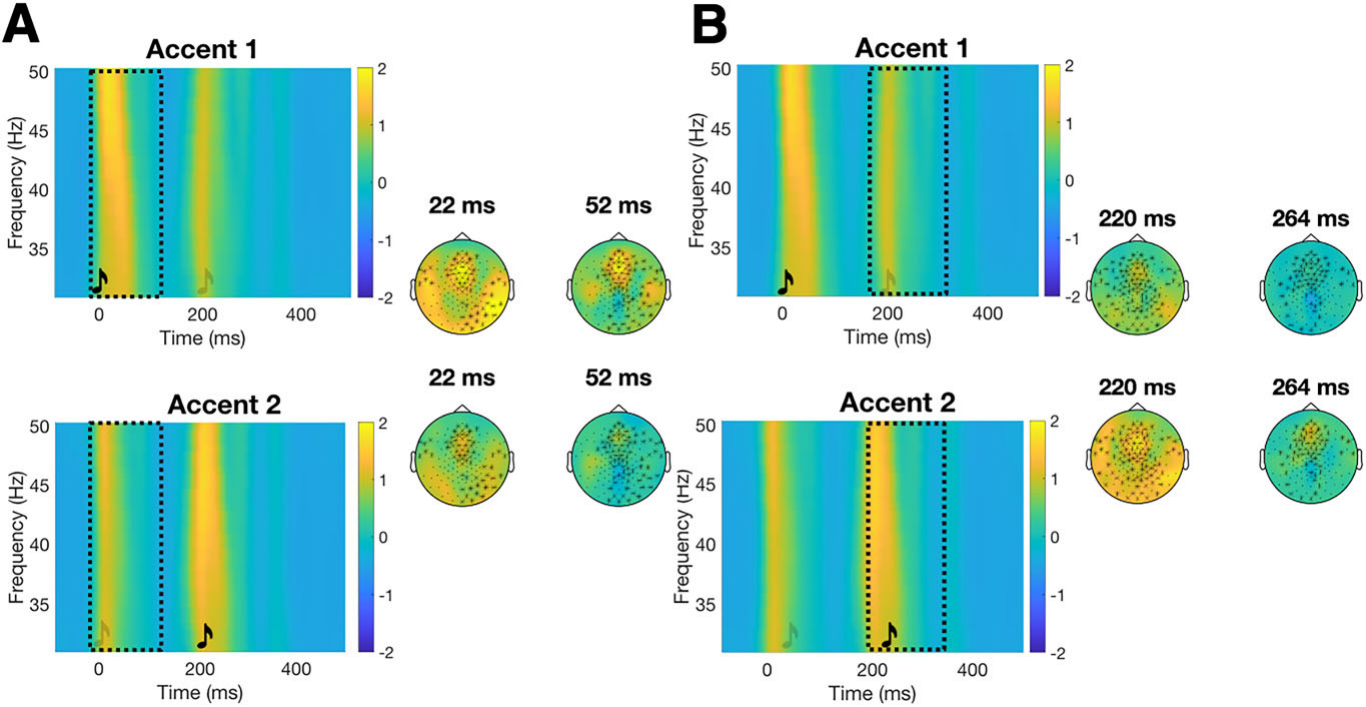
Accented-Beat1 (***A***) and Accented-Beat2 (***B***) gamma effects. TFRs and topographies for gamma neural activity at the first tone (***A***, Accented-Beat1 effect) and at the second tone (***B***, Accented-Beat2 effect). See Figure 1 for a description of the representation. Increased gamma neural activity was found for the first tone when this was accented [in the Accent1 condition (top row) as compared with the Accent2 condition (bottom row)] in ***A***. Increased gamma neural activity was found for the second tone when this was accented [in the Accent2 condition (bottom row) as compared with the Accent1 condition (top row)] in ***B***.

Results from the cluster-based permutation tests revealed increased beta power to the tones when they were physically accented compared with when they were not. This effect appeared both at the position of the first (Accented-Beat1 effect; *p *< 0.001) and the second tone (Accented-Beat2 effect; *p *= 0.002) primarily on central channels (Fig. 2, Table 1). There was an increase also in gamma power to the tones when they were physically accented compared with when they were not at the position of the first (Accented-Beat1 effect) and the second tone (Accented-Beat2 effect; both *p *< 0.001) on frontocentral channels (Fig. 3, Table 1).

**Table 1.** Summary of significant differences between conditions

Comparison	Effect	Frequency band	Time window, ms	Regional distribution	*p*
Accent1 vs Accent2	Accented-Beat1 effect	Beta	0–144	Central	<0.001
		Gamma	2–120	Frontocentral	<0.001
Accent1 vs Accent2	Accented-Beat2 effect	Beta	188–340	Central	0.002
		Gamma	188–316	Frontocentral	<0.001
Inferred1 vs Inferred2	Inferred-Beat2 Inverted effect	Beta	176–370	Right parietal	0.013

**Figure 4.**
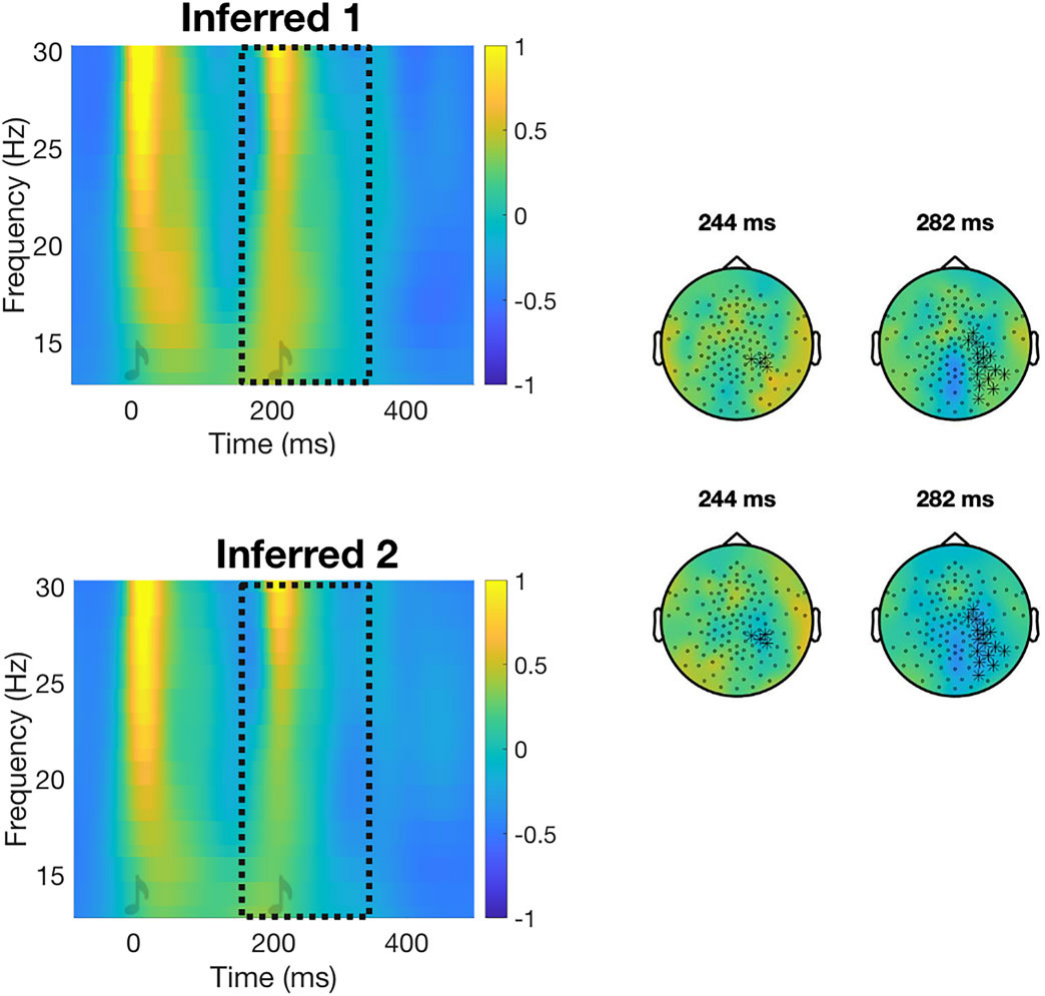
Inferred-Beat2 Inverted beta effect. TFRs and topographies for EEG evoked beta neural activity. See Figure 1 for a description of the representation. We found increased beta neural activity around the onset of the second tone in the Inferred1 condition (inferred beat on the first tone) compared with the Inferred2 condition (inferred beat on the second tone). This is opposite to what we expected and what we found in the accented conditions.

When comparing the two inferred beat conditions, we found a significant cluster at the onset of the second tone, with greater beta power in the Inferred1 condition (inferred beat on the first tone) compared with the Inferred2 condition (inferred beat on the second tone) in the lower right parietal area. This is contrary to what we would have anticipated, which would have been greater beta power in the Inferred2 compared with the Inferred1 condition at the second tone, given that the second tone was accented in the Inferred2 condition (Inferred-Beat2 Inverted effect; *p *= 0.013, Table 1). There were no other significant differences in beta or gamma power between the two inferred beat conditions (Fig. 4).

### Associations of neural measures with self-reported and behavioral measures

We performed Pearson’s correlations to investigate associations of individual differences in neural correlates of beat processing with individual differences in musicality and a behavioral measure of beat processing. Table 2 reports the descriptive statistics for all self-reported and behavioral measures. We calculated the neural indexes of beat processing based on the results of the cluster analysis. For each significant cluster, we calculated the mean power difference between the two conditions using the channels, time range, and frequency range of the corresponding cluster. Since we found five significant clusters, we used five indexes of neural correlates of beat processing: accented beat processing beta power difference at the first tone (Beta-Accented-Beat1) and at the second tone (Beta-Accented-Beat2), accented beat processing gamma power difference at the first tone (Gamma-Accented-Beat1) and at the second tone (Gamma-Accented-Beat2), and inferred beat processing beta power difference at the second tone (Beta-Inferred-Beat2).

**Table 2.** Descriptive statistics for behavioral measures

	Mean (SD)
Gold-MSI (*N *= 57)	
Perceptual abilities	46.90 (8.84)
Musical training	22.42 (11.21)
General sophistication	71.68 (21.39)
BBA (*N *= 52)	
Simple *d*’	1.78 (0.59)
Complex *d*’	1.53 (0.67)
Overall *d*’	1.65 (0.51)

Scores for general sophistication can range from 18 to 126. Scores for the perceptual abilities subscale range from 9 to 63. Scores for the musical training subscale range from 7 to 49.

#### Associations between neural measures and self-reported musicality

**Figure 5.**
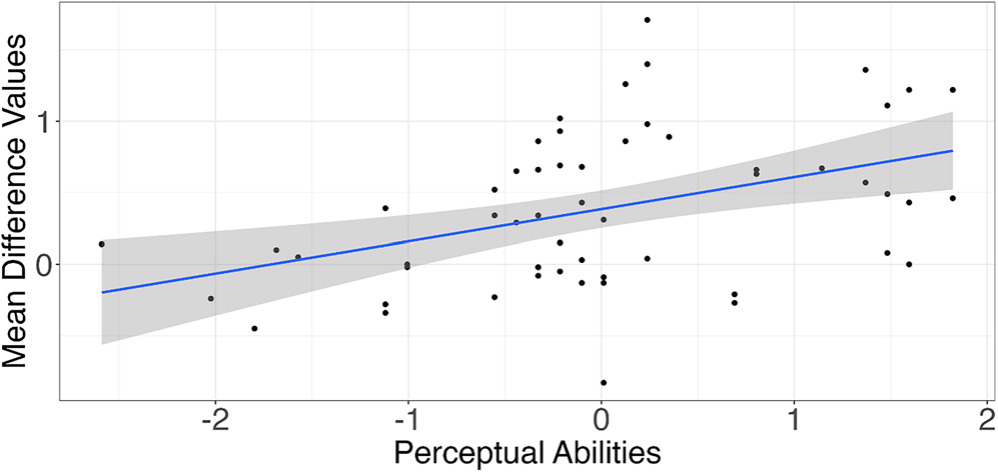
Significant correlation between power values of the Accented-Beat1 cluster in the beta frequency band with the Gold-MSI Perceptual Abilities Subscale.

The Beta-Accented-Beat1 effect was associated with higher musicality scores in perceptual abilities (*r *= 0.42, *p *= 0.001, Fig. 5), musical training (*r *= 0.28, *p *= 0.037), and general sophistication (*r *= 0.36, *p *= 0.007) in the expected direction. Additionally, the Gamma-Accented-Beat2 effect was negatively associated with lower musicality scores in perceptual abilities (*r *= −0.27, *p *= 0.045), musical training (*r *= −0.32, *p *= 0.017), and general sophistication (*r *= −0.33, *p *= 0.012). See Table 3 for all correlations between the neural correlates of beat processing and musicality. Only the correlation between the Beta-Accented-Beat1 effect and perceptual abilities remained significant at a Bonferroni-corrected alpha level (0.05/15 = 0.003, Fig. 5).

**Table 3.** Correlations between the Gold-MSI and neural correlates of beat processing

Cluster	Frequency	Gold-MSI		
		Perceptual abilities	Musical training	General sophistication
Inferred-Beat2 Inverted Effect	Beta	*r *= 0.01, *p *= 0.920	*r *= 0.08, *p *= 0.538	*r *= 0.10, *p *= 0.460
Accented-Beat2 Effect	Beta	*r *= 0.07, *p *= 0.627	*r *= −0.07, *p *= 0.595	*r *= 0.04, *p *= 0.795
Accented-Beat1 Effect	Beta	***r *= 0.42, *p *= 0.001**	*r *= 0.28, *p *= 0.037	*r *= 0.36, *p *= 0.007
Accented-Beat1 Effect	Gamma	*r *= 0.20, *p *= 0.136	*r *= 0.09, *p *= 0.516	*r *= 0.16, *p *= 0.245
Accented-Beat2 Effect	Gamma	*r *= −0.27, *p *= 0.045	*r *= −0.32, *p *= 0.017	*r *= −0.33, *p *= 0.012

The correlation that remained significant after Bonferroni correction is marked in bold.

We examined if this association remained significant after accounting for individual differences in age and socioeconomic status as well as in biological sex with a multiple linear regression model with the Accented-Beat1 effect as the dependent variable and perceptual abilities and the covariates as independent variables. The association remained significant after adding covariates (Table 4).

**Table 4.** Association between the Beta-Accented-Beat1 effect and perceptual abilities after controlling for covariates

Measure	Β	SE	95% CI	*p*
Perceptual abilities	0.33	0.119	(0.096–0.564)	0.007
Age	−0.16	0.118	(−0.391 to 0.071)	0.180
Sex	1.21	0.453	(0.326–2.094)	0.010
SES	−0.15	0.117	(−0.379 to 0.079)	0.198
*R* ^2^	0.31			<0.001

#### Associations between neural and behavioral measures

Pearson’s correlation analyses on the neural indices of beat processing and behavioral rhythm task scores (simple *d*’, complex *d*’, and overall *d*’) did not reveal any significant correlations (all *p*’s > 0.05).

One potential explanation for the lack of observed associations between the neural and behavioral correlates of beat processing may be the previously unknown test–retest reliability of the rhythm discrimination task. To evaluate this explanation, we conducted a follow-up study to assess the test–retest reliability of the beat-based advantage task. The study involved *N* = 27 participants (mean age = 19.7 years, SD = 4.2 years), who completed the task twice on the same online platform used in the main study remotely, with an approximate interval of 2 weeks between sessions (mean interval = 2.2 weeks, SD = 0.4). The results indicate a moderate test–retest reliability (*r *= 0.58, *p *= 0.002). Thus, it is unlikely that the neural correlates of beat processing were not correlated with the rhythm discrimination performance solely due to the test–retest reliability of the beat-based advantage task.

## Discussion

The present study examined the neural underpinnings of spontaneous beat processing and its relationship with self-reported musicality as well as with rhythm discrimination ability. Our findings suggest that even without active attention to the rhythmic stimuli, individuals demonstrate distinct neural responses to the beat when it is acoustically marked, in particular in the beta and gamma frequency bands. These markers were associated with individuals’ self-reported musicality. The neural processing of ambiguous stimuli, however, does not follow a previously acoustically marked beat pattern if individuals are not explicitly instructed to continue to imagine the corresponding beat pattern.

Increased response to physically accented beats in the beta and the gamma frequency band is consistent with previous work with the same stimuli and procedures (Kasdan et al., 2022; Persici et al., 2023), with the same stimuli but with an instruction to pay attention to the stimuli (Iversen et al., 2009) and with studies showing beat processing even in the absence of attention (Bouwer et al., 2016). The beta frequency band has been proposed to be involved in hierarchical timing, internalized timing information, and beat perception (Fujioka et al., 2012, 2015; Cirelli et al., 2014). Specifically, it has been suggested that periodic bursts in beta oscillations drive periodic expectations about incoming sensory inputs, supporting the processing of temporally regular stimuli. The gamma band region is primarily involved in sensory cue selection (Merchant et al., 2015) and in the formation of temporal expectancies (Zanto et al., 2005, 2006; Fujioka et al., 2009). Our results thus indicate that beta and gamma oscillations have a similar role in spontaneous beat processing as when the participants are explicitly instructed to pay attention to the beat.

We did not observe, however, the same beat-related increase in oscillatory activity in the case of inferred beats in contrast to a previous study using the same stimuli but with the instruction to continue to imagine the beat even when the physical accent was not present anymore (Iversen et al., 2009).

There are a few possibilities for the lack of the expected effects in the case of the inferred conditions. One possibility is that participants failed to anticipate the beat due to a lack of attention to the stimuli; however, we do not believe this to be the case because neural responses in the physically accented conditions were similar to prior work. Another possibility is that participants failed to implicitly learn the beat pattern at the beginning of the block, and therefore did not establish a representation of the expected beat pattern. The latter view is consistent with studies showing less efficient implicit extraction of regularities in adults compared with infants or children (Nemeth et al., 2013; Rohlf et al., 2017; Mueller et al., 2018). In line with this idea, previous work with infants has found evidence for inferred beat processing (Flaten et al., 2022).

While we did not observe the expected beat-related increase in the inferred conditions, we found an inverted effect at the second tone. This result, together with the visual inspection of Figure 4, indicates that in ambiguous beat patterns without a physically accented beat, participants placed the beat on the first tone when the physical accent appeared at the second tone at the beginning of the block. However, they showed no clear preference for a beat pattern when the physical accent appeared at the first tone at the beginning of the block. This finding is contrary to our hypotheses and hard to interpret. If participants simply failed to anticipate the beat in the present paradigm due to task-related factors in the inferred conditions, then we would not have observed any effect in the inferred conditions. Placing the beat on the first tone, even when the physical accent appeared at the second tone at the beginning of the block, could suggest a general preference for the beat at the first tone. In this case, however, we would have expected an increased beta activity at the first tone and also when the physical accent appeared at the first tone at the beginning of the block. Therefore, we believe this inverted effect lacks a clear interpretation and could be a spurious finding. Future research should investigate the perception of ambiguous rhythmic patterns by systematically manipulating attention to better understand the role of perceptual bias under different circumstances. Based on the current results, it appears that participants may need explicit instructions in the absence of clear sensory cues in order to maintain a certain beat pattern.

We investigated the relationship between individual differences in the neural correlates of beat processing and self-reported musicality as well as rhythm discrimination ability, to determine if there are shared mechanisms underlying these characteristics. While individual differences studies using behavioral measures are quite common, studies using neural measures (e.g., EEG, MRI, fMRI) typically focus on investigating phenomena at the group level by averaging data from participants. Deriving neural indices of cognitive processes at the individual level could be important for several reasons. It is becoming evident that musical abilities show a broad distribution in the population and studies focusing on individual differences in brain mechanisms could help to better understand the neural underpinnings of this diversity, as well as age-related changes at the behavioral level. Research on individual differences can also illuminate how this individual variability relates to genetic variations (Dubois and Adolphs, 2016; Mosing et al., 2016; Niarchou et al., 2022; Lopez et al., 2023). A better understanding of individual differences and age-related changes in musicality can also clarify the mechanisms underlying the associations between musicality and other cognitive abilities such as linguistic and motor abilities. Beat processing has been linked to language and motor abilities by several previous studies (Hill, 2001; Kotz and Schwartze, 2010; Arnal and Giraud, 2012; Arnal et al., 2015; Gordon et al., 2015; Assaneo et al., 2019; Morillon et al., 2019; Canette et al., 2020; Poeppel and Assaneo, 2020; Swaminathan and Schellenberg, 2020; Ladányi et al., 2023). A better understanding of the relationships between various aspects of musicality and other cognitive processes can improve music-based treatments for various conditions such as developmental language disorder, dyslexia, attention-deficit hyperactivity disorder, autism spectrum disorder, Parkinson's disease, and Alzheimer's disease.

Following the individual differences approach, we demonstrated that increased neural response to the beat at the onset of the sequence was related to higher self-reported musical perceptual skills. This finding indicates that individuals with better musicality are more attuned to rhythm and timing, allowing them to predict and prepare for beats. The relationship observed only with the beta and not with the gamma frequency band suggests that beat processing is more relevant to musicality than the processing of acoustic characteristics of the stimuli. Furthermore, this relationship was found for the neural correlate of beat processing at the position of the first tone, but not the second. According to rhythmic grouping principles, when two unaccented tones are followed by a rest, the second tone is typically perceived as accented, forming a weak-strong-rest pattern (Povel and Okkerman, 1981). Therefore, beat processing mechanisms might be more engaged when the accent occurs at the first position, making it a more sensitive measure of beat processing and leading to a correlation with perceptual abilities.

Contrary to our predictions, the neural correlates of beat processing were not associated with the performance on the rhythm discrimination task, which can be considered a behavioral measure of beat processing. Based on our follow-up study, this cannot be explained by the test–retest reliability of the rhythm discrimination task. Several factors may account for the lack of this relationship. It is possible that the two tasks are sensitive to different aspects of rhythm processing (see (Fiveash et al. 2022) on the multidimensionality of rhythmic ability). For example, the rhythm discrimination task has a strong working memory component, whereas the EEG task only has minimal working memory demands. Additionally, in the rhythm discrimination task, participants are instructed to pay attention to the stimuli, while the beat processing EEG task involves passive listening without explicit instruction to pay attention. Moreover, the rhythm discrimination task features more complex rhythmic patterns than the beat processing EEG task, making beat extraction more challenging. As a result, it is possible that the two tasks are assessing different constructs of beat processing, and individual differences in task performance may arise from distinct underlying factors.

Note that our sample predominantly consisted of female participants, due to reasons unrelated to the research question (we tested the accompanying parents of infants participating in another experiment, and these parents were primarily females). Future studies should aim for a sample more representative of the general population.

In summary, we have shown that increased activity in the beta and gamma frequency ranges is linked with the spontaneous processing of physically accented beats. These neural markers were associated with individuals’ self-reported perceptual abilities indicating distinct neural mechanisms in individuals with different perceptual abilities. Our study motivates future work on the relationship between the neural markers of beat processing and behavioral correlates of musicality.

## Synthesis

Reviewing Editor: Luke Remage-Healey, University of Massachusetts Amherst Decisions are customarily a result of the Reviewing Editor and the peer reviewers coming together and discussing their recommendations until a consensus is reached. When revisions are invited, a fact-based synthesis statement explaining their decision and outlining what is needed to prepare a revision will be listed below. The following reviewer(s) agreed to reveal their identity: NONE.

Reviewer 1: Thanks to the authors for considering the comments. The manuscript is now more clear, and the authors responded appropriately to my comments.

Reviewer 2: The revised manuscript includes all the previous recommendations and presents a refined exploration of neural mechanisms underlying beat processing using EEG data in the context of music perception. The authors have effectively addressed several methodological issues and provided further clarity in their experimental design, specifically in the Beat Perception EEG task and Beat-Based Advantage task.

The authors made corrections to the figures and the legends match with that of the figures. In the revised version, the explanation of the inferred beat condition and the neural response associated with it is clearer. However, the beta activity for Beat 2 in the inferred condition is still somewhat counterintuitive. The authors should elaborate on whether the task structure or potential task-related factors (such as impaired anticipation) could account for these findings.

The authors notably improved the discussion of beta band increase in relation stimulus novelty and periodic expectations.

The correlation between neural markers of beat processing and behavioral task scores remains weak, and the authors attribute this to issues of test-retest reliability in the rhythm discrimination task. While this is a plausible explanation, the paper could benefit from discussing other potential factors that might influence these results, such as the complexity of rhythm discrimination or individual differences in task performance.

The paper is now clearer and stronger in its methodology and could make a valuable contribution to the field once the above concerns are addressed.

## Author Response

We thank the reviewers for their kind words and are pleased that they found our modifications satisfactory. We also appreciate their additional suggestions. Below, you will find our responses to the comments in bold, with the corresponding changes in italics. In the manuscript, we have marked the changes from the previous version in bold.

Reviewer 1: Thanks to the authors for considering the comments. The manuscript is now more clear, and the authors responded appropriately to my comments.

Reviewer 2: The revised manuscript includes all the previous recommendations and presents a refined exploration of neural mechanisms underlying beat processing using EEG data in the context of music perception. The authors have effectively addressed several methodological issues and provided further clarity in their experimental design, specifically in the Beat Perception EEG task and Beat-Based Advantage task.

The authors made corrections to the figures and the legends match with that of the figures. In the revised version, the explanation of the inferred beat condition and the neural response associated with it is clearer.

1. However, the beta activity for Beat 2 in the inferred condition is still somewhat counterintuitive. The authors should elaborate on whether the task structure or potential task-related factors (such as impaired anticipation) could account for these findings.

We thank the reviewer for the comment. We agree that the beat activity observed for Beat 2 when the physical accent was presented at the position of Beat 1 at the beginning of the block is counterintuitive. We do not believe that the task structure or task-related factors could explain this result. We think it's more likely that this is a spurious finding. We have now elaborated on this point in the manuscript. We have also slightly modified the structure and moved this point after discussing the potential reasons for the lack of the expected increase in beta activity to the beat in the inferred conditions (Page 17-18).

While we did not observe the expected beat-related increase in the inferred conditions, we found an inverted effect at the second tone. This indicates that, in ambiguous beat patterns without a physically accented beat, participants placed the beat on the first tone when the physical accent appeared at the second tone at the beginning of the block. However, they showed no clear preference for a beat pattern when the physical accent appeared at the first tone at the beginning of the block. This finding is contrary to our hypotheses and hard to interpret. If participants simply failed to anticipate the beat in the present paradigm due to task-related factors in the inferred conditions, then we would not have observed any effect in the inferred conditions. Placing the beat on the first tone, even when the physical accent appeared at the second tone at the beginning of the block, could suggest a general preference for the beat at the first tone. In this case, however, we would have expected an increased beta activity at the first tone also when the physical accent appeared at the first tone at the beginning of the block. Therefore, we believe this inverted effect lacks a clear interpretation and could be a spurious finding. Future research should investigate the perception of ambiguous rhythmic patterns by systematically manipulating attention to better understand the role of perceptual bias under different circumstances. Based on the current results, it appears that participants may need explicit instructions in the absence of clear sensory cues in order to maintain a certain beat pattern.

The authors notably improved the discussion of beta band increase in relation stimulus novelty and periodic expectations.

2. The correlation between neural markers of beat processing and behavioral task scores remains weak, and the authors attribute this to issues of test-retest reliability in the rhythm discrimination task. While this is a plausible explanation, the paper could benefit from discussing other potential factors that might influence these results, such as the complexity of rhythm discrimination or individual differences in task performance.

We thank the reviewer for pointing out that the discussion of factors contributing to the lack of association between the EEG markers and behavioral beat processing tasks was not sufficiently clear. We have now edited this section (Page 19-20).

Contrary to our predictions, the neural correlates of beat processing were not associated with the performance on the rhythm discrimination task, which can be considered a behavioral measure of beat processing. Based on our follow-up study, this cannot be explained by the test-retest reliability of the rhythm discrimination task. Several factors may account for the lack of this relationship. It is possible that the two tasks are sensitive to different aspects of rhythm processing (see Fiveash et al. (2022) on the multidimensionality of rhythmic ability). For example, the rhythm discrimination task has a strong working memory component, whereas the EEG task only has minimal working memory demands. Additionally, in the rhythm discrimination task participants are instructed to pay attention to the stimuli, while the beat processing EEG task involves passive listening without an explicit instruction to pay attention. Moreover, the rhythm discrimination task features more complex rhythmic patterns than the beat processing EEG task, making beat extraction more challenging. As a result, it is possible that the two tasks are assessing different constructs of beat processing, and individual differences in the task performance may arise from distinct underlying factors.

The paper is now clearer and stronger in its methodology and could make a valuable contribution to the field once the above concerns are addressed.
